# Vectored immunoprophylaxis: an emerging adjunct to traditional vaccination

**DOI:** 10.1186/s40794-017-0046-0

**Published:** 2017-02-10

**Authors:** John W. Sanders, Todd A. Ponzio

**Affiliations:** 1 Wake Forest University School of Medicine, Medical Center Blvd, Winston-Salem, NC 27157 USA; 2 Salisbury Veterans Affairs Medical Center, Salisbury, NC USA; 3 Naval Medical Research Center, 503 Robert Grant Ave, Silver Spring, MD 20910 USA; 4 Section on Infectious Diseases and Department of Bio-Engineering, Wake Forest University School of Medicine, Winston-Salem, USA

**Keywords:** Vectored immunoprophylaxis, Immunoprophylaxis by gene transfer, Vector-mediated antibody gene transfer, Gene therapy, Vector, Broadly neutralizing antibody, Salivary gland, HIV, Vaccine

## Abstract

The successful development of effective vaccines has been elusive for many of the world’s most important infectious diseases. Additionally, much of the population, such as the aged or immunocompromised, are unable to mount an effective immunologic response for existing vaccines. Vectored Immunoprophylaxis (VIP) is a novel approach designed to address these challenges. Rather than utilizing an antigen to trigger a response from the host’s immune system as is normally done with traditional vaccines, VIP genetically engineers the production of tailored antibodies from non-hematopoietic cells, bypassing the humoral immune system. Direct administration of genes encoding for neutralizing antibodies has proven to be effective in both preventing and treating several infectious diseases in animal models. While, a significant amount of work has focused on HIV, including an ongoing clinical trial, the approach has also been shown to be effective for malaria, dengue, hepatitis C, influenza, and more. In addition to presenting itself as a potentially efficient approach to solving long-standing vaccine challenges, the approach may be the best, if not only, method to vaccinate immunocompromised individuals. Many issues still need to be addressed, including which tissue(s) makes the most suitable platform, which vector(s) are most efficient at transducing the platform tissue used to secrete the antibodies, and what are the long-term effects of such a treatment. Here we provide a brief overview of this approach, and its potential application in treating some of the world’s most intractable infectious diseases.

## Background

From the early practice of scarification to prevent smallpox through the creation of targeted, recombinant vaccines, the development of effective vaccines has been one of the great achievements in public health and medicine, resulting in millions of lives saved. Modern vaccines typically protect by eliciting immunity following exposure to an inactivated or attenuated whole pathogen or recombinant components of a pathogen [1]. This approach works well for diseases in which natural infection leads to immunity and protection against re-infection and has resulted in the eradication of smallpox and dramatic declines in such diseases as diphtheria, measles, and polio [2]. However, it has been more challenging to develop effective vaccines against diseases for which prior infection does not offer full future protection, such as HIV, malaria, hepatitis C virus, and influenza A [1].

Although cellular immunity is certainly important, humoral immunity appears to play the most significant role in the protection associated with most vaccines [3]. Passive immunization achieved through the infusion of serum has played a significant historical role in the treatment and prevention of infection [4, 5]. The recent development of hybridoma technology and humanized monoclonal antibodies have resulted in a new class of antibody-based drugs with demonstrated and potential efficacy in cancer, inflammatory diseases, addiction, and infectious diseases [6]. Within this context, there has been an increased interest in passive immunization utilizing monoclonal antibodies produced in plants or transgenic animals for infections such as Ebola virus and MERS-CoV [7, 8]. However, logistical requirements including the need for high antibody concentrations requiring repeated injections due to the short half-life of antibodies, a cold-chain for delivery, and trained medical personnel for delivery create potential limitations to the use of this therapy, especially in low resource areas [1, 9]. The development of passive immunization by gene therapy could be a solution to some of those logistical issues and holds potential promise as either an adjunct to standard vaccination in populations who do not generate a sufficient immune response or for pathogens able to evade current vaccination strategies due to antigenic variability.

**Fig. 1:**
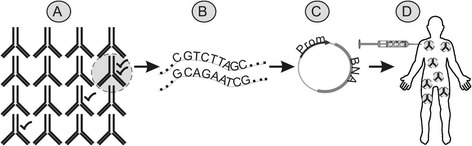
Immunoprotection by vectored immunoprophylaxis (VIP). **a** Identification of an effective broadly-neutralizing antibody (BNA). The most potent of the BNAs is noted with two *checkmarks* in the graphic. **b** The genetic sequences of the antibody variable regions are determined. **c** The genetic sequence for the BNA can then be placed downstream from an appropriate promoter (Prom) within a suitable vector. **d** The vector can then be administered to the subject in an appropriate tissue platform, such as muscle. The BNA produced by the vector and secreted by the tissue confers the host subject with broad and lasting protection from the targeted pathogen

Originally proposed as a concept in 2002 [10], passive immunization by vector-mediated delivery of genes encoding broadly neutralizing antibodies for in vivo expression has been referred to as Immunoprophylaxis by Gene Transfer (IGT) [11], Vector-Mediated Antibody Gene transfer [11], or Vectored Immunoprophylaxis (VIP) [6, 12]; and for sake of consistency, ‘VIP’ is used here. Rather than passively transfering pre-formed antibodies, VIP is a process in which genes encoding previously characterized neutralizing antibodies are vectored into non-hematopoietic cells which then secrete the monoclonal antibodes encoded by those genes [1] (See Fig. 1.) This vectored delivery and production of specified antibodies allows for protection without generating a standard immune response and results in endogenous antibody production that has the potential to be sustained [9]. The approach has several benefits, including: 1) it does not require the host have the ability to respond immunologically, 2) the antibody can naturally be selected for a specific pathogen targets, as well as specific epitopes, 3) the antibody can be genetically modified to further enhance its activity, and, 4) vectors can be selected or engineered to have tropic characteristics targeting specific tissues and cells, potentially allowing either systemic or enhanced localized antibody production [9].

### Infections for which VIP has been tested

VIP has been demonstrated to be effective in a host of animal models for the prevention of infection with several pathogens, especially those commonly afflicting travelers (see Table 1), including influenza A virus [13, 14], malaria (*Plasmodium falciparum*) [15], hepatitis C virus [16], respiratory syncitial virus [17], *Bacillus anthracis* [18], dengue virus [19], and chickungunya virus [20]. In addition to the protection conferred by systemic neutralizing antibodies, protection against infection with influenza A virus has also been demonstrated following intranasal administration of vectored local antibody production [21].

**Table 1:** Infections for which vectored immunoprophylaxis has been tested

	Vector	Animal models	References
HIV (or SHIV)	Plasmid, lentivirus, rAAV2, scAAV2, rAAV8, scAAV1,	BALB/c mice, NSG mice, Rag-1 mice, Rhesus macaques	[12, 23–26, 29, 32, 33]
hepatitis C virus	rAAV9	Rosa26-Fluc mice	[16]
*Plasmodium falciparum*	rAAV8	C57BL/6 mice	[15]
influenza A virus	Ad5, rAAV8, rAAV9	BALB/c mice, NSG mice, ferret, rhesus macaques	[13, 14, 21]
respiratory syncytial virus (RSV)	Ad5, rAAVrh10	BALB/c mice	[17]
*Bacillus anthracis*	Ad5, rAAVrh10	C57BL/6 mice	[18]
dengue virus	Plasmid	C57BL/6 mice	[19]
chikungunya virus	Plasmid	BALB/c mice	[20]

Adapted from [1]

*Abbreviations*: *rAAV* recombinant adeno-associated virus, *scAAV* self-complementary adeno-associated virus, *Ad5* adenovirus serotype 5

By far, the most extensive and promising exploration of VIP for an infectious disease has been against HIV. In the initial study demonstrating the potential of VIP, a recombinant adeno-associated virus (rAAV) vector using a dual-promoter system generated both light and heavy chains of IgG1b12, one of the early broadly neutralizing antibodies described for HIV. The rAAV was injected into the quadricep muscles of immunodeficient mice and biologically active antibody was found in sera for over 6 months [10]. This study provided the first evidence that rAAV vectors could transfer antibody genes to muscle, and muscle tissue was a suitable platform to produce and distribute the antibodies throughout the circulation [11]. Follow-on studies used a native macaque SIV gp120-specific Fab molecule as an immunoadhesin, a chimeric, antibody-like molecules that combine the functional domain of a binding protein with immunoglobulin constant domains, which were considered to be superior to single chain (scFv) or whole antibody (IgG) molecules with respect to achievable steady-state serum concentrations [22]. Six of nine rhesus macaques were completely protected against intravenous challenge with virulent SIV and still had stable immunoadhesin levels 6 years after injection [11]. The three subjects not protected were found to have developed an immune response to the immunoadhesin by 3 weeks after injection [11].

Another group used an rAAV vector injected into the quadriceps muscle of a humanized mouse to express an array of broadly neutralizing antibodies: 2G12, IgG1b12, 2F5, 4E10 and VRC01. Though VRC01 serum levels as low as 8.3 μg/mL provided protection from an intravenous challenge with HIV, they achieved concentrations as high as 100 μg/mL for at least 12 months [12]. They followed-up that study by optimizing the broadly neutralizing antibody, and although muscle was chosen as a platform for expression and secretion of the IgG1 isotype, antibodies were found to effectively reach the vaginal mucosa. Animals receiving VIP that expressed a modified VRC07 antibody (concentration of nearly 100 μg/ml in the serum and 1 μg/ml in vaginal wash fluid) were completely resistant to repetitive intravaginal challenge by a heterosexually transmitted founder HIV strain [23].

Saunders, et al., used an rAAV serotype 8 vector to produce a full length IgG of a simianized form of the broadly neutralizing antibody VRC07 in macaques which was protective against simian-human immunodeficiency virus (SHIV) infection 5.5 weeks after treatment [24]. SHIVs are chimeric viruses constructed to express the HIV envelope glycoprotein to be used in vaccine experiments to evaluate neutralizing antibodies. The antibody reached levels up to 66 μg/ml for 16 weeks, but immune suppression with cyclosporine was needed to sustain expression due to the development of anti-idiotypic antibodies [24].

The approach to preventing HIV was enhanced further by fusing the immunoadhesin form of CD4-Ig with a small CCR5-mimetic sulfopeptide at the carboxy-terminus (eCD4-Ig). eCD4-Ig is more potent than the best broadly neutralizing antiody and binds avidly to the HIV-1 envelope glycoprotein. Rhesus macaques expressed 17–77 μg/mL of fully functional rhesus eCD4-Ig for more than 40 weeks after injection with a self complimentary serotype 1 AAV (scAAV1) vector and were completely protected from multiple challenges with a simian/human immunodeficiency virus, SHIV-AD8 [25]. Of note, the rhesus eCD4-Ig was also markedly less immunogenic than rhesus forms of four well-characterized broadly neutralizing antibodies [25].

In addition to disease prevention as noted above, studies have also demonstrated an application for VIP in the effective treatment of previously-infected animals. Using HIV-1-infected humanized mice, Horwitz, et al., demonstrated that following initial treatment with anti-retroviral therapy (ART), a single injection of adeno-associated virus directing expression of broadly neutralizing antibody 10-1074, produced durable viremic control after the ART was stopped [26].

The first human trial using the VIP approach started in January 2014 and is a phase 1, randomized, blinded, dose-escalation study of an rAAV1 vector coding for PG9, a potent broadly neutralizing antibody, in high risk, healthy adult males (ClinicalTrials.gov number, NCT01937455). Another study evaluating using VIP in HIV-positive subjects is scheduled to get underway soon [6].

### Vectors

Many options exist for vectoring the transgene into the host tissue, each with distinct advantages and limitations. Naked plasmid DNA is relatively easy to use, does not elicit significant immunogenicity, and has the potential for inexpensive large-scale production [20, 27]. Recent advances in both the mechanism of delivery [28] and optimization of plasmid and electroporation conditions [29] have improved the concentration and duration of antibody production, but it has yet to prove as potent as viral vectoring.

Viral vectors offer the advantage of efficient, rapid delivery of the transgene into host cells and the potential for integration into the host genome, allowing for sustained expression [1]. The life cycle of a virus consists of attachment, penetration, uncoating, replication, gene expression, assembly and budding. Replication and gene expression typically take place in the nucleus where viral genomes persist episomally or integrate into the host genome (i.e., a provirus). Vectors that persist episomally can provide sustained transgene expression in post-mitotic tissue, but since they do not alter the host genome, they may be lost if and when the cells divide. Vectors that integrate into to the host genome may provide life-long transgene expression in dividing cells but could also lead to insertional mutagenesis resulting in apoptosis or malignant transformation [30].

Adenoviral vectors produce rapid, but transient, gene expression that could be ideal for responding to a disease outbreak, but would have limitations for long term protection [1]. Adenovirus serotype 5 (Ad5) has successfully transduced protective antibodies for respiratory syncytial virus (RSV), influenza A virus (IAV), and *Bacillus anthracis* [14, 17, 18]. The Ad5 genome is easy to engineer and remains episomal, but there is significant pre-existing immunity to Ad5, estimated at 50% of the adult population worldwide and even higher in sub-Saharan Arica, which decreases the ability to transfer the transgene. Additionally, it can result in systemic cytokine release creating a sepsis presentation and there is significant tissue tropism for the liver when delivered intravenously. Alternative adenoviral vectors are being researched [31].

Lentivral vectors are better suited for long term expression since they typically integrate into the genome and can transduce dividing and non-dividing cells. They have successfully been utilized to transduce hematopoietic stem cells to produce broadly neutralizing antibodies against HIV in mouse models [32, 33]. However, because they can integrate into the host genome, there is concern for mutagenesis. Newer generation lentiviral vectors contain deletions in their long-terminal repeat (LTR) and a self-inactivating (SIN) LTR, leaving them replication incompetent, which should make them much safer, but this question is not fully answered [30].

Although other viral vectors are being explored, rAAV vectors are currently the favored vehicle for delivering the antiody genes into the host tissue due to their efficiency in gene transfer [34]. In contrast to other viral vectors, such as adenovirus, rAAV’s have not been associated with any human diseases and do not stimulate signficant immunologic reaction, and are therefore able to induce long-term expression of non-self-proteins [34]. They are engineered to consist of the antibody gene expression cassette flanked by the AAV ITRs (inverted terminal repeats), which are the only part of the AAV genome present in the rAAV vector and are required for rAAV vector genome replication and packaging. Despite a relativley small packaging capacity of 5 kb, both heavy- and light-chain antibody genes can be incorporated into a single vector, either using a promoter for each gene cassette or a single promoter for expression with the heavy and light chain separated by a foot-and-mouth disease virus 2A peptide [9].

### ‘Immunization’ site selection

All studies to date have targeted skeletal muscle as the platform for transfection and antibody production. Muscle offers some significant advantages. It is easily accessible for localized vector administration, and some muscle groups can be removed in the event of mutagenesis or auto-immunity without functional consequence. However, muscle has certain disadvantages as well. It is a tissue that does not normally produce circulating proteins and therefore may not do it efficiently. It also contains antigen-presenting dendritic cells that could induce immune responses which might eliminate transduced cells or induce auto-immunity. Additionally, the removal of muscle tissue would likely have a significant effect on a subject’s lifestyle in the event of a potential unexpected VIP-induced pathology.

Other platforms have been considered. For example, some authors have suggested the liver as an alternative site [35]. Unlike muscle, it is designed to secrete circulating proteins. It is also thought to be less immunogenic. However, transduction would require systemic administration of the vector, and there would be no simple means of eliminating expression in the event of a complication. Another potential site could be the salivary glands. While it is well-know that the salivary glands secrete proteins into the oral cavity, it may be less well appreciated that they have also been used as a platform to deliver therapeutic proteins, including the IgG Fc fragment and a host of other proteins, into the systemic circulation [36, 37]. Transgenes delivered to the salivary gland tend to favor being sorted either into the saliva or the blood, though it is currently a challenge to predict which direction a particular protein will sort [7]. The major paired salivary glands are also easily accessible, and the parotid glands are encapsulated, which minimizes vector spillage into the general circulation. Futhermore, in the event of complications, the transfected glands could be removed without creating major disability.

### Potential safety issues

Safety concerns associated with VIP include genotoxic events typically associated with any viral vector mediated gene therapy, such as inflammation, a random insertion disrupting normal genes, activation of proto-oncogenes, and insertional mutagenesis [38]. There are many factors which affect the likelihood of developing a genotoxic event including the vector, the targeted insertion site, the transgene, the targeted cell type, and host factors including age and underlying disease [39]. The risk of genotoxicity or carcinogenicity can potentially be decreased by selection of the promoter and the integration site, using novel techniques such as Clustered Regularly Interspaced Short Palindromic Repeat (CRISPR)-Cas9 (an RNA-guided gene-editing platform that allows for cutting of DNA in a specified gene), but much more work needs to be done to better characterize safety and efficacy of these methods [39].

As the purpose of VIP is to produce a monoclonal antibody, the possibility of producing a paraproteinemia similar to that caused by multiple myeloma, other hematologic malignancies, primary amyloidosis, or a monoclonal gammopathy of undertermined significance (MGUS) is a concern. The most benign of these is MGUS, but it has been increasingly recognized to have pathologic associations including is nephropathy secondary to monoclonal gammopathy of renal significance (MGRS), neuropathy, oculopathy, and dermopathy as well as possible associations with autoimmunity and coagulopathy and an epidemiologic association with early mortality from a variety of apparently unrelated causes [40–42]. Any of these conditions could result from a monoclonal gammopathy produced by VIP. However, it should be noted that MGUS is very common, occurring in 3% of the population older than 50 years old, and most of these associations remain either unclear or uncommon [40]. However, the potential for autoimmunity should be of particular concern. It is possible that the monoclonal antibody could interact with self-antigen and either stimulate an autoimmune antibody that interacts with self-antigen [40] or neutralizes the intended effect of the monoclonal antibody.

## Conclusion

Vectored Immunoprophylaxis has demonstrated great promise in a variety of pre-clinical studies as a potential adjunct to vaccination in patients not able to respond effectively to immunization or as an alternative to vaccination for infectious diseases not effectively covered by current vaccines. The rapid identification of specific neutralizing antibodies is likely to increase the potential for this method. One could imagine uses for VIP such as an adjunct to vaccination for influenza in the elderly and immunocompromised, for HIV protection in high risk populations, or as part of a ring vaccination strategy in an outbreak of a disease such as Ebola. Many important questions remain, including the ability to produce equally effective clinical results in human trials, the duration of response, and the potential for side-effects. Mutagenesis at the site of transfection is a common concern, but the development of an immune response to the transgene product or the off-target binding of the antibodies are more likely scenarios, either of which could result in decreased efficacy of the procedure or a significant auto-immune reaction. Questions also remain concerning the best vector and the optimal tissue site for transfection. Despite these questions and concerns, the advantages offered in settings ranging from chronic protection of the aged or immunocompromised to rapid protection for early responders in the event of a bioterror or emerging infection event are significant and intriguing. Further pre-clinical and clinical studies are certainly warranted.

## Abbreviations

Ad5: Adenovirus serotype 5
ART: Anti-retroviral therapy
HIV: Human immunodeficiency virus
IAV: Influenza A virus
IGT: Immunoprophylaxis by gene transfer
ITR: Inverted terminal repeats
LTR: Long-terminal repeat
rAAV: Recombinant adeno-associated virus
RSV: Respiratory syncytial virus
scFv: Single chain antibody
SHIV: Simian-human immunodeficiency virus
SIN: Self-inactivating LTR
SIV: Simian immunodeficiency virus
VIP: Vectored immunoprophylaxis
